# Predictors of weight progression among HIV infected adults on anti-retroviral treatment in Mekelle hospital, Tigray, Ethiopia: A longitudinal study

**DOI:** 10.1371/journal.pone.0327392

**Published:** 2025-07-18

**Authors:** Ataklti Kiflu Arefayne, Hagazi Gebre Meles, Goitom Halefom Senbete, Kibrom Birhane Gebrelibanos, Letemichael Mezgebo Gebrekorkos, Yohannes Kinfe Gebreyohannes

**Affiliations:** 1 Department of Biostatistics, School of Public Health, College of Health Sciences, Mekelle University, Mekelle, Tigray, Ethiopia; 2 Department of Epidemiology and Biostatistics, School of Public Health, College of Health Sciences, Aksum University, Aksum, Tigray, Ethiopia; University of Oulu: Oulun Yliopisto, FINLAND

## Abstract

**Background:**

Human immunodeficiency virus (HIV) remains a significant global public health issue, causing over 35 million deaths until 2017. In 2013, 24.7 million people in sub-Saharan Africa were living with HIV with 1.5 million new infections and 1.1 million deaths. Evidences on weight progression among HIV patients in low resource settings are limited. Additionally, many of the previously conducted studies were cross-sectional. Therefore, this study aimed to examine predictors of weight progression among adult HIV infected patients on ART from January 2017 to December 2019 in Mekelle hospital, Tigray, Ethiopia.

**Methods:**

Retrospective longitudinal study was conducted from March 2, 2020 to March 14, 2020 at Mekelle General Hospital, Tigray, Ethiopia. A total of 97 individuals were followed every six months for two years. STATA version15 was used for data analysis. Linear mixed model was used to examine progression of weight after the baseline measurement. Regression coefficients of the final model and their 95% confidence intervals were used as measures of association with p-value below 0.05 indicating statistical significance.

**Results:**

Baseline median age of participants was found to be 35 years (IQR = 27.5 to 40). At baseline, 39.18% were underweight, 53.61% normal and 7.22% overweight. About one-fifth (19.59%) of the study subjects were diagnosed with tuberculosis infection. Patients who had normal weight at baseline lost 1.89 kg (β = −1.89, 95% CI: −3.11, −0.66) at 6 months compared to underweight patients. Patients with CD4 level of >350 cells/µL at baseline lost 2.53 kg (β = −2.53, 95% CI: −4.43, −0.63) weight lower than patients with CD4 level of <=350 cells/µL, at 24 months.

**Conclusion and recommendation:**

From this study, it can be concluded that weight of participants is influenced by measurement and individual level factors implying the need to address measurement and individual level factors when dealing with weight progression.

## Introduction

Obesity has tripled globally since 1975 indicating a global increase in weight status. Body mass index (BMI) is an index of weight for height that is commonly used to categorize weight status in adults as underweight, normal weight, overweight, and obese [1,2]. Human immunodeficiency virus (HIV), is a viral infection, that causes immune-deficiency and Acquired Immunodeficiency Syndrome (AIDS), characterized by severe clinical symptoms, including weight loss [3]. Weight loss during pre-Antiretroviral Therapy (ART) is linked to HIV disease progression and development of opportunistic infections, while unintentional weight loss can be caused by depression, hyperthyroidism, oral health issues, street drug use, and infections [4,5]. ART slows the body’s production of HIV making HIV a chronic and manageable illness with increased rate of obesity [2,6-8]. ART reduces mortality rates in HIV patients, improving weight and lean body mass, especially in those with greater pre-treatment immunological and virological compromise [9].

HIV remains a significant global public health issue, causing over 35 million deaths until 2017. In 2017 only, 940,000 people died of HIV-related causes globally [10]. In 2013, 24.7 million people in sub-Saharan Africa were living with HIV with 1.5 million new infections and 1.1 million deaths [11]. The First evidence of HIV epidemic in Ethiopia was notified in 1984, onwards resulting in millions of deaths and hundreds of thousands of orphans [9]. ART began in Ethiopia in 2003, and free ART was launched in 2005. About 769,500 Ethiopians are currently living with HIV, of whom all require ART and 392, 086 are currently taking the treatment [12].

Regardless of the major progresses made in response, HIV epidemics pose global public health threats, with 22 million out of 37 million living with HIV at the end of 2014 not accessing ART [13]. Despite the availability of effective HIV prevention tools and antiretroviral therapy, there has been insufficient progress in reducing new HIV infections globally in which growing number are noted to be overweight or obese, paralleling the global epidemic of obesity [1,2,14,15].

Cohort analysis, a crucial part of ART patient monitoring, compares baseline characteristics with 6 and 12 months followed by yearly measurements, revealing improvements in Cluster of Differentiation (CD4) count and weight. The median CD4 count for a group of patients is a good measure of immunosuppression and a predictor of mortality and serious opportunistic infections [16].

ART initiation in developed countries is consistently linked to weight gain, particularly in patients with lower BMI or CD4 + T cell count [6,17–20]. It is unclear whether change of therapy results in reversal of weight gain [10]. Assessment of BMI in sub-Saharan Africa showed 49% obesity and 32% overweight among HIV infected women of black African origin [15]. HIV-induced weight loss is a significant predictor of poor prognosis and contributes to undernutrition in HIV-infected individuals in low socio-economic societies [21]. Previously, in the HIV pandemic, the concern of weight progression was weight loss. While in the current developments in HIV treatments, weight gain occur as part of weight progression [22].

HIV infection has been linked to body composition, ranging from muscle wasting to lipodystrophy. Nowadays, lipodystrophy is observed prominently among adults who have aged with HIV and were treated with older ART regimens, and even with contemporary ART regimens [23]. Routine viral load and CD4 lymphocyte count monitoring is often unavailable or inaccessible in resource-limited settings, hindering the study of weight progression in low socio-economic contexts [24].

Studies conducted previously were mainly in developed countries, hence little is known in low socio-economic settings. Besides, previous studies were mainly cross-sectional studies, making associations doubtful. Using a repeated measures design increases power, improves efficiency and allows testing a time × treatment interaction [25]. In this study data were approached through linear mixed model. Few studies have been conducted in Ethiopia while no other study has been conducted to assess predictors of weight progression among HIV infected individuals in Tigray. Therefore, this study has aimed to fill the information gap of weight progression by determining the weight progression over treatment time and identifying the key factors affecting it by employing linear mixed model.

## Methods and materials

### Study area and period

The study was conducted at Mekelle General Hospital which is located in Mekelle City, the capital of Tigray Regional state, Ethiopia. It has been built and started service officially, in July, 1962, with an intention to serve for about 20,000 populations, living in and around the city catchment area. Since the last 20 years its service provision has dramatically been increasing in response to the rapid urbanization expansion of the city and increasing population density.

Mekelle General Hospital has been serving as a referral hospital for the peoples of the Tigray regional state, and for some other districts of the neighboring regional states of Afar and Amhara. Currently it provides Clinical services for about 1.2 million peoples of both the local & neighboring districts. The hospital gives services focusing on HIV care such as voluntary testing and counseling, provider-initiated testing and counselling, prevention of mother to child transmission and ART services in a clinic to both physician and self-referred patients. In 2010 Ethiopian Fiscal Year (EFY) reports on HIV care shows a total of 7237 individuals ever started on ART and 6430 were aged 15 and above, 4511 currently on ART with 4218 aged 15 and above, and 237 were newly started on ART in which 213 were aged 15 and above [26]. Data was extracted from March 2, 2020 to March 14, 2020.

### Study design

A retrospective longitudinal study was conducted employing quantitative methods.

### Population

#### Source population.

The source population was all adult HIV positive patients enrolled on ART care from January 2017 to December 2019 at Mekelle General Hospital.

#### Study population.

The study population was all adult HIV positive patients who were followed every 6 months for 2 years that were enrolled on ART care from January 2017 and December 2019 in Mekelle General Hospital.

#### Study unit.

Selected adult HIV infected individuals on ART care in Mekelle General Hospital

### Eligibility criteria

#### Inclusion criteria.

All newly enrolled HIV positive aged 15 and above in Mekelle General Hospital from January 2017 to December 2019 were included in the study.

#### Exclusion criteria:.

- Pregnant mothers and transfer outs were excluded from this study.
- Those who have no at least 2 measurements of the outcome variable were also excluded from the study.

### Sample size and sampling procedure

The sample size needed to achieve the desired power should be determined, and the largest sample size was considered. Moreover, 95% CI, 80% power, 1 mean scale factor and 1 variability scale factors were used to calculate the sample size. Collecting repeated measurements can simultaneously increase statistical power for detecting changes while reducing the costs of conducting a study. In spite of the advantages over cross-sectional designs, repeated measures designs complicate the crucial process of selecting a sample size. Studies with independent observations, repeated measurements taken from the same participant are correlated, and the correlations must be considered in calculating the appropriate sample size.

Some current software packages used for sample size calculations are based on oversimplified assumptions about correlation patterns. It is recommended to use the program GLIMMPSE (URL:http://glimmpse.samplesizeshop.org/) for computing sample size for repeated measures and longitudinal designs [25,27]. Using the GLIMMPSE web-based software, the minimum final sample size with desired power of 80% is 86 at each measurement and adding 10% contingency the total minimum sample becomes 95. But the total population in the hospital with in the study period were 213, and 19 of them were excluded due to exclusion criteria. Study populations were ordered chronologically using their respective medical record numbers starting from 1 up to 194; then systematic random sampling was applied to get the study participants, the sampling interval was 194/95 ≈ 2, and lottery method was used to select the first participant and 97 participants were selected, having at most 5 measurements for each individual & 463 observation were collected.

In stating the hypothesis for calculating the sample size, the main effect of time was used and the effect size used was 0.5. In our case, the variance of difference (intraclass correlation) between baseline weight and weight at 6 month was 0.93 in a previous study [28]. This variance of difference can be directly used as an estimate for the variances of the weight measures, Var(weight). As for the required correlations, 10 correlation values need to be estimated since there are 5 repeated measurements. Unstructured pattern of correlation between measurements was assumed.

### Study variables

#### Dependent variable.

- Weight progression (in kilograms) change over time (whether it is increase or decrease): weight changes from baseline were assessed at 6 months, 12 months, 18 months and 24 months.

#### Independent variables.

**Measurement level variables**

- Age in years
- WHO clinical staging at enrollment (stage 1, stage 2, stage 3, or stage 4)
- Tuberculosis (TB) infection status at Enrollment
- Functional status
- Opportunistic Infections (OI)
- Cotrimoxazole Prophylactic Therapy (CPT) adherence
- Antiretroviral (ARV) adherence
- ARV drug
- Isoniazid Prophylactic Therapy (IPT) prophylaxis
- Length of stay on ART

**Individual level variables**

- Sex (male or female)
- Viral load at 6 months
- Baseline BMI
- CD4 count
- Hemoglobin
- Occupational status

### Operational definitions

**HIV Cohort:** all HIV patients that starts ART at the same month [16].

**ART Cohort analysis:** compares baseline characteristics of ART start-up groups (monthly cohorts) with their status at 6 and 12 months then yearly. Key indicators for the clinical and district teams to see how well the program is doing, such as the percentage of patients still on a first-line regimen or able to work at 6 and 12 months, are calculated using this report. It allows the teams, in a meaningful way, to compare success at 6 and 12 months of ART with earlier or later cohorts, or with other districts [16].

**Currently on ART:** Number of adults and children currently receiving antiretroviral therapy (ART) [29].

**Ever started on ART:** Number of adults and children started ART since start of ART program [16].

**Immuno-compromised:** or immunodeficiency is a condition in which the immune system’s capability to defend infectious disease and cancer is compromised or entirely absent. Most cases of immunodeficiency are secondary to extrinsic factors that affect the patient’s immune system. In the clinical setting, the immunosuppression by some drugs, such as steroids, can be either an adverse effect or the intended purpose of the treatment [9].

**Lipodystrophy syndromes**: are a group of genetic or acquired abnormalities in which the body is unable to produce and maintain healthy fat tissue. The medical condition is characterized by abnormal or degenerative states of the body’s adipose tissue. A more specific term, lip-atrophy is used when describing the loss of fat from one area (usually the face) [30].

### Data collection and quality control

A standard abstraction sheet was used to extract the data from patients’ cards. This form is developed using the standardized ART entry and follow up form employed by the ART clinics in Ethiopia. Three advanced ART nurses who were trained on comprehensive HIV care and treatment services and were involved in patient follow ups at the time of the study were collected the data. Data collection was supervised by the principal investigator. All data collection forms were examined for clarity and consistency. Weight (kg) which was measured every six months by the ART nurses were recorded at each interval for four consecutive intervals (5 measurements). Weight measurements were taken using calibrated digital scales, under minimal clothing. Measurements were performed by ART trained health professionals who followed a predefined protocol to ensure uniformity across participants and time points. In addition to the weight, BMI was also calculated at each measurement interval for each study participants. Full information maximum likelihood estimation were used to handle missing observations in the analysis of the data set [31].

### Data analysis

Descriptive statistics such as mean, standard deviation, tables and graphs were used to describe the characteristics of the participants. STATA version 15 was used for analysis. Linear regression was used to characterize and screen data for problems of multi-collinearity. Linear Mixed Model was used to examine changes in weight after the baseline measurement. Model fit was examined using Akaike information criterion (AIC) of the competing models. Regression coefficients of the final model and their 95% confidence intervals were used to measure magnitude and direction of the association between the predictors and outcome variable. A p-value of less than 0.05 was considered statistically significant.

#### Linear mixed modeling.

In longitudinal or panel data, each unit is observed at several occasions over time. This makes it possible to study individual change, either due to the passage of time or due to explanatory variables [32]. In addition to this, repeated observations of a given individual are not independent and hence violate the assumption, independence of observations, which is required by most statistical analysis techniques.

Linear mixed model have many advantages over cross-sectional studies namely: have growth curves that are different for each individual, handling unbalanced number of repeated measures across subjects, the covariance’s between the repeated measures can be modeled as well, simple to add higher levels, in the case of balanced data we can use ANOVA based analysis of *f* or *t* tests, and it is straightforward to include time-varying or time-constant explanatory variables to the model [33,34].

#### Types of factors and their related factors in Linear Mixed Models (LMM).

**Fixed Factors**: defined as a categorical or classification variable, for which the investigator has included all levels (or conditions) that are of interest in the study. Fixed factors might include qualitative covariates; or ordinal classification variables in an observational study. Levels of a fixed factor are chosen so that they represent specific conditions, and they can be used to define contrasts (or sets of contrasts) of interest in the research study.

**Random Factors**: A random factor is a classification variable with levels that can be thought of as being randomly sampled from a population of levels being studied. All possible levels of the random factor are not present in the data set, but it is the researcher’s intention to make inferences about the entire population of levels. The classification variables that identify the Level 2 and Level 3 units in repeated-measures/longitudinal data sets are often considered to be random factors. Random factors are considered in an analysis so that variation in the dependent variable across levels of the random factors can be assessed, and the results of the data analysis can be generalized to a greater population of levels of the random factor.

**Specification of LMMs**: The general specification of an LMM presented in this study refers to a model for a longitudinal two-level data set, with the first index, *t*, being used to indicate a time point, and the second index, *i*, being used for subjects [35–41].

Here in this study time (t_i_) was from 0 up to 4; and n_i_ was from 1 up to 97.

**Bivariate Linear Mixed Regression Analysis:** Potential candidate variables were identified by developing bivariate linear mixed regression. In this stage, the presence of an association between the outcome variable and each explanatory variable was investigated without controlling the effect of the other explanatory variables. Those explanatory variables which were significant at 75% confidence level (with p-value of 0.25) in the bivariate analysis were entered to the Multivariable for adjustment [33].

#### Multivariable linear mixed regression analysis.

This model was constructed by entering all variables with p-value of ≤ 0.25 in the bivariate mixed linear regression analysis. Those variables with p-value of < 0.05 in this analysis stage were declared statistically significant and results reported with regression coefficients, and their respective 95% CI. Four models containing the respective candidate variables were fitted.

**Model I (null model)**: This is a model that was run without any explanatory variables to test

the within individual change variability on weight. Being the first step in Linear Mixed Model, this intercept only model is important to decide whether the data is fit for linear mixed modeling or not. Because the model enables us to calculate the Intra-class Correlation Coefficient (ICC) – the percentage of variability explained by the upper level (individual).

ICC was calculated using the formula:


$$ICC=\frac{between\;individual\;variance}{between\;individual\;variance+within\;individual\;variance}\;$$ (1)

The formula for this model is:


$$Y_{it}\;=\;\beta_{0t}\;+\;u_{0t}$$ (2)

Where:

- Yit is the outcome variable that is weight of i^th^ participant (i = 1…97) measured at time t (0 month, 6 month, 12 month, 18 month or 24 month)
- β_0t_ is the intercept that is the mean weight of study participants
- u_0t_ is an individual level residual or error.

**Model II (model with measurement level factors only):** measurement level variables that were significant at the bivariate analysis were entered to this Multivariable analysis for

adjustment. In this model, the contribution of each measurement level factor on weight progression was explored. An ICC was also calculated and compared with the ICC

calculated from the null model. To explore the relative contribution in explaining weight, Proportional Change in Variance (PCV) was calculated in reference to the null model.

The formula for this is:$PCV=\frac{vo-vi}{vo}$

Where: v_o_ is variance in the null model and

v_i_ is variance in the consecutive models

The formula for this model is: $Y_{it}\;=\;\gamma_{00}\;+\;\gamma_{p0}X_{pit}\;+\;u_{0t}\;+\;e_{ij}\backslash )$

Where:

- γ_00_ is the intercept
- γ_p0_ is the regression coefficient
- Xp is the measurement level explanatory variable, p = number of measurement level variables
- u_0t_ is the individual level error
- e_it_ is the measurement level error
- The subscripts i and t represent for the individual and measurement level respectively.

**Model III (model with individual level factors only):** individual level variables which became significant at the bivariate analysis were entered to this Multivariable analysis for adjustment. In this model, the contribution of each individual level factor on weight was explored. ICC was calculated and compared with that of the null model. To explore the relative contribution of the individual level factors in explaining weight, PCV was calculated.

The formula for this model is:


$$Y_{it}\;=\;\gamma_{00}\;+\;\gamma_{0q}Z_{qt}\;+\;u_{0t}\;+\;e_{it}\mathrm{\backslash ]}$$ (3)

Where:

- γ_00_ is the intercept, γ_0q_ is the regression coefficient.
- Zq is the individual level explanatory variable; u_0t_ is the individual level error
- e_it_ is the measurement level error

The subscripts i and t represent for the individual level and for the measurement respectively.

**Model IV (mixed model with measurement and individual level factors):** It is a multivariable analysis model adjustment of both measurement and individual level variables which were statistically significant in their respective models. The formula for this mixed model is:


$$Y_{ij}\;=\;\beta_{0}\;+\;u_{i0}\;+(\beta_{1}\;+\;u_{i1})t_{ij}\;+\;e_{ij}\mathrm{\backslash ]}$$ (4)

Where:

• β_0_ + β_1_ are fixed effects.
• u_i_ are the random effects and not associated with covariates
• U_i_= (u_i0_ + u_i0_) then u_i_ ~ N(0, G), i= 1,2,3…97, j= 0,1,…4
• Y_ij_ be the weight measurement for patient i at observation j

The model has fixed or deterministic part and the random part.

#### Parameter estimation.

The fixed-effects (measures of association) estimate the associations between weight change and the various explanatory variables expressed as regression coefficients with their 95% CIs. The Maximum Likelihood (ML) estimation method was used to estimate the parameters. Whereas the intercept only random-effects (measures of variation) were reported as an ICC which is the proportion of individual level variance as compared to the total variance. PCV expresses the change in variance between the null model (Model I) and the consecutive models [34].

#### Model diagnostics multicollinearity and interaction.

The presence of multi-collinearity among independent variables was checked using Variance Inflation Factor (VIF) at cut off point of 10. Predictors with a VIF value of less than 10 indicate absence of multi-collinearity [42]. Interactions between individual and measurement level factors were assessed for significant interactions.

#### Model selection.

AIC was used to compare and select the model that best fits the data. The model that has the lowest AIC value from all candidate models was considered as the model that best fits the data [43].

### Ethical clearance

The study was employed after ethical approval was obtained from ethical review committee of Mekelle University College of Health Sciences with approval and supporting letter (Ref. ERC 1593 2020). A supporting letter from Tigray Regional Health Bureau was given to Mekelle General Hospital, then permission to use the ART follow up data was obtained from the hospital administration. Being secondary data, taking informed consent from original data subjects is not applicable for this study. However, individual identifiers were kept confidentially. Accessed data was used for the purpose of this study only and was and will not be shared with other third persons.

## Results

### Descriptive statistics

Data were collected from 97 individuals on ART in Mekelle General Hospital that were retrospectively followed for 2 years and measured at 6 months interval ranging from 3 to 5 measurements for each individual. Data were collected from a total of 463 observations nested within 97 individuals. Regarding follow-up time, 100% participant records were reviewed at baseline, six month and one year, 96.91% at 18th month, 80.41% at 24th month records were reviewed. The mean weight of the study participants at baseline, 6 month, 12 month, 18 month and 24 month were 50.37 kg (SD = 0.84), 53.45 kg (SD = 0.77), 53.95 kg(SD = 0.82), 54.54 kg (SD = 0.87), and 54.87 (SD = 1.01) respectively.

Among the participants 77(79.38%) were females and 64(65.98%) of the participants were unemployed. The median age of participants at baseline was found to be 34 years and 50% of the respondents were in the age group between 27 and 40 years. About one fifth (19.59%) of the participants had no formal education. Majority (86.6%) of the participants were urban residents. At the time of ART initiation, 38 participants (39.18%) were underweight, 52 (53.61%) were normal weight, 7 (7.22%) were overweight and no one were obese at baseline measurement. Almost One fifth (19.59%) of the study subjects were diagnosed with tuberculosis infection, regarding WHO clinical staging 51(52.58%), 16(16.49%), 17(17.53%), and 13(13.40%) were stage I, stage II, stage III, and stage IV respectively. Of the study subjects, 68(70.10%) were working, 18(18.56%) ambulatory and 11(11.34%) bed ridden (Table 1).

**Table 1:** Measurement and individual level characteristics of Human Immunodeficiency Virus (HIV) infected patients on anti-retroviral treatment in Mekelle hospital, Tigray, Ethiopia from January 2017 to December 2019 (n = 97 observations = 463).

Measurement level factors							
		Time of measurement					
		Baseline		6 months	12 months	18 months	24 months
Age	Median (IQR)	34 (27-40)		34.5 (27.5-40.5)	35 (28-41)	36 (28.5-41.5)	37 (28-42)
BMI	UW (<18.5)	38 (39.18)		22 (22.68)	17 (17.53)	17 (18.09)	13 (16.67)
	NW(18.5–24.9)	52 (53.61)		67 (69.07)	72 (74.23)	69 (73.40)	56 (71.79)
	OW(25-29-9)	7 (7.22)		6 (6.19)	7 (7.22)	7 (7.45)	8 (10.26)
	Obese (>=30)	0		2 (2.06)	1 (1.03)	1 (1.06)	1 (1.28)
TB infection	No	78 (80.41)		78 (80.41)	92 (94.85)	91 (96.81)	76 (97.44)
	Yes	19 (19.59)		19 (19.59)	5 (5.15)	3 (3.19)	2 (2.56)
WHO Clinical stage	Stage I	51(52.58)		59(60.82)	87(89.69)	88(93.62)	76(97.44)
	Stage II	16(16.49)		13(13.40)	3(3.09)	1(1.06)	2(2.56)
	Stage III	17(17.53)		20(20.62)	6(6.19)	4(4.26)	
	Stage IV	13(13.40)		5(5.15)	1(1.03)	1(1.06)	
Functional status	Working	68(70.10)		84(86.60)	94(96.91)	90(95.74)	75(96.15)
	Ambulatory	18(18.56)		8(8.25)	2(2.06)	3(3.19)	2(2.56)
	Bed ridden	11(11.34)		5(5.15)	1(1.03)	1(1.06)	1(1.28)
Tuberculosis prophylaxis	Yes	1(1.03)		66(68.04)	68(70.10)	68(72.34)	57(73.08)
	No	96(98.97)		31(31.96)	29(29.90)	26(27.66)	21(26.92)
OIs	No	73(75.26)		75(77.32)	90(92.78)	89(94.68)	76(97.44)
	Yes	24(24.74)		22(22.68)	7(7.22)	5(5.32)	2(2.56)
Pain assessment result	No pain	77(79.38)		80(82.47)	91(93.81)	89(94.68)	75(96.15)
	WHO step 1	0		2(2.06)	1(1.03)	1(1.03)	1(1.28)
	WHO step 2	10(10.31)		7(7.22)	1(1.03)	0	0
	WHO step 3	10(10.31)		8(8.25)	4(4.12)	4(4.26)	2(2.56)
Use of other medications	Yes	7(7.22)		6(6.19)	6(6.19)	6(6.38)	5(6.410
	No	90(92.78)		91(93.81)	91(93.81)	88(93.62)	73(93.59)
ARV adherence	Good			90(92.78)	88(90.720	91(96.81)	75(96.15)
	Fair			1(1.03)	1(1.03)	0	0
	Poor			6(6.19)	8(8.25)	3(3.19)	3(3.85)
**Individual level characteristics at baseline**							
Socio-demographic characteristics			Number (%)				
Sex	Male		20 (20.62)				
	Female		77 (79.38)				
Residence	Urban		84 (86.6)				
	Rural		13 (13.4)				
Occupational status	Employed		33 (34.02)				
	Unemployed		64 (65.98)				
Educational status	No education		19 (19.59)				
	Primary		46 (47.42)				
	Secondary		21 (21.65)				
	Tertiary		11 (11.34)				
Religion	Orthodox		94 (96.91)				
	Muslim		1 (1.03)				
	Catholic		2 (2.06)				
Six-month Viral load	Low		89 (91.75)				
	High		8 (8.25)				

Key: ARV – Antiretroviral; BMI – Body Mass Index; IQR – Inter-quartile Range; OIs – Opportunistic Infections; TB – Tuberculosis; WHO – World Health Organization

### Exploratory data analysis

#### Individual weight profile plot of participants over time.

**Fig 1:**
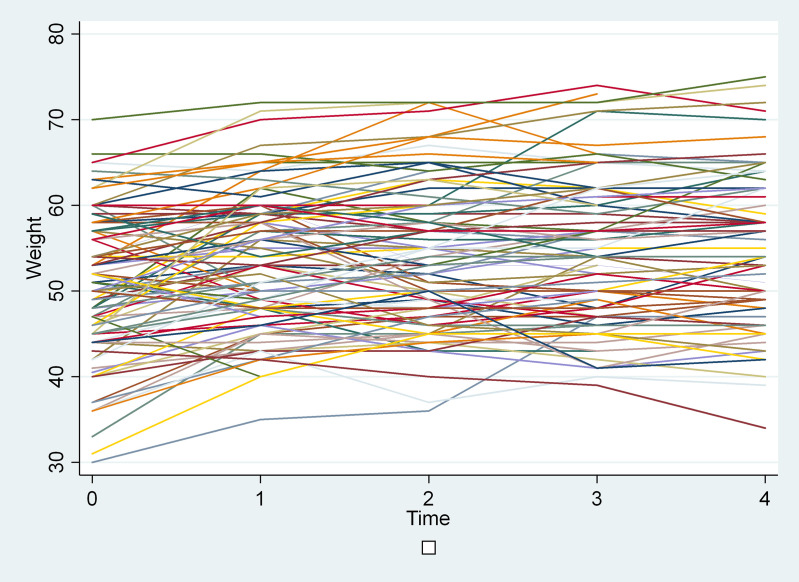
Individual weight over time plots of patients on antiretroviral treatment in Mekelle hospital, Tigray, Ethiopia. Legend = each line represents one study subject.

The graph for the progression of weight among individuals shows that the weight of individuals over time is varrying between individuals and in some individuals weight gradually increased whereas in other individuals the weight decreased. Every individual has its own intercept as can be seen from the graph (Fig 1).

#### Mean weight profile plot.

**Fig 2:**
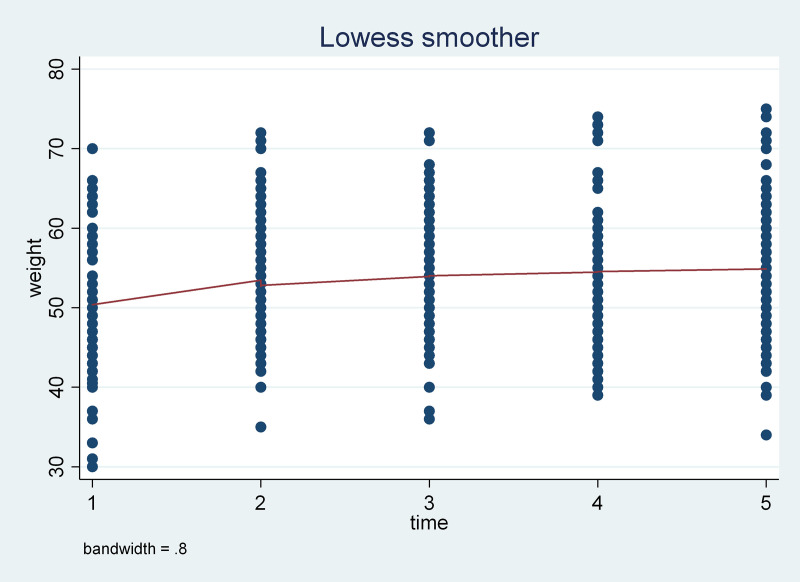
Mean weight plot over time of patients on antiretroviral treatment in Mekelle hospital, Tigray, Ethiopia.

The mean of the participants increased from 50.38 kg (95% CI: 48.73, 52.03) at baseline to 53.45 kg (95% CI: 51.91, 55.00) at six months, to 53.95 kg (95% CI: 52.34, 55.56) at 12 months, to 54.54 kg (95% CI: 52.84, 56.24) at 18 months, and to 54.87 kg (955 CI: 52.90, 6.85) at 24 months (Fig 2).

### Bivariate linear mixed model analysis results

All measurement level variables except CPT adherence, ARV side effects and clients’ HIV prevention plan were with p-value ≤ 0.25. Sex, religion, residence and viral load at six months become with p-value ≤ 0.25 and hence entered into the multivariable analysis.

### Multivariable linear mixed model analysis

This multivariable linear mixed regression was built by entering the variables that have a p-value ≤ 0.25 at the bivariate analysis.

#### Random effects results.

**Model 1: Null model**: In this model the output showed that there was significant variation in participants’ weights across individuals (v_w_ = 56.31, p < 0.001). The ICC which is.8960 to mean 89.60% of the total variation in weight is attributed to individual difference (Table 2).

**Table 2:** Measurement and individual level factors associated with weight change among Human Immunodeficiency Virus (HIV) infected patients on anti-retroviral treatment in Mekelle hospital, Tigray, Ethiopia from January 2017 to December 2019 (n = 97 observations = 463).

Variables		Null model	Measurement level variables only model	Individual level variables only model	Mixed model
**Fixed effects estimate**					
		**Estimate (95% CI)**	**Estimate (95% CI)**	**Estimate (95% CI)**	**Estimate (95% CI)**
Intercept		51.63 (50.09, 53.18)***			
Age	15-24		Ref		Ref
	25-34		2.51 (−0.82, 5.83)		1.51 (−0.93, 3.94)
	35-44		3.71 (−0.06, 7.47)		2.86 (0.29, 5.43)*
	45-54		8.39 (3.01, 13.77)		4.38 (0.63, 8.12)*
	55-64		5.45 (−2.25, 13.16)		2.20 (−3.82, 8.21)
	64^+^		14.56 (0.24, 28.87)		6.42 (2.24, 15.07)
Time	Baseline		Ref		
	6 month		3.83 (2.55, 5.11)		4.33 (3.13, 5.53)***
	12 month		3.74 (2.26, 5.22)		4.69 (3.29, 6.09)***
	18 month		4.17 (2.53, 5.81)		5.17 (3.57, 6.78)***
	24 month		4.69 (2.82, 6.59)		5.72 (3.84, 7.61)***
Functional status	Working		Ref		Ref
	Ambulatory		−1.30 (−2.87, 0.27)		0.20 (−3.23, 3.62)
	Bed ridden		−1.94 (−4.34, 0.45)		2.86 (−4.17, 9.88)
WHO clinical stage	Stage I		Ref		Ref
	Stage II		−1.24 (−2.84, 0.36)		0.40 (−1.25, 2.05)
	Stage III		−0.19 (−1.99, 1.61)		2.08 (−0.96, 5.11)
	Stage IV		−1.88 (−4.04, 0.25)		1.47 (−3.60, 6.54)
Tuberculosis infection status	Yes		Ref		Ref
	No		−2.25 (−5.01, 0.56)		−12.04 (−21.92, −2.16)*
Tuberculosis prophylaxis status	Yes		Ref		Ref
	No		1.50 (0.09, 2.91)		−8.00 (−13.24, −2.76)**
TB status and TB prophylaxis Interaction			4.41 (−0.97, 9.79)		6.23 (1.06, 11.40)*
Opportunistic infections	No		Ref		Ref
	Yes		1.45 (−1.68, 4.59)		1.27 (−1.73, 4.26)
Pain assessment	No pain		Ref		Ref
	WHO step I		−2.20 (−6.42, 2.02)		−3.66 (−7.89, 0.57)
	WHO step II		−1.04 (−3.80, 1.73)		−2.55 (−5.11, 0.01)
	WHO step III		−0.12 (−3.20, 2.96)		−0.74 (−3.62, 2.15)
Other medications	Yes		Ref		Ref
	No		3.52 (0.26, 6.78)		3.00 (0.14, 5.87)*
ARV drug adherence	Good		Ref		
	Fair		−2.02 (−6.85,2.81)		−2.35 (−7.08, 2.39)
	Poor		−2.69 (−4.64, −0.73)		−1.46 (−3.35, 0.43)
Sex	Male			Ref	Ref
	Female			−5.94 (−8.00, −3.87)	−5.20 (−7.40, −3.00)***
Religion	Orthodox			Ref	Ref
	Muslim			−0.83 (−8.88, 7.22)	−0.72 (−8.83, 7.38)
	Catholic			−3.43 (−9.37, 2.49)	−2.20 (−8.43, 4.03)
Viral load	>1000 copies			Ref	Ref
	<=1000 copies			−0.14 (−3.16, 2.870	0.53 (−2.59, 3.64)
CD4 count	>=350			Ref	Ref
	<350			−0.70 (−2.59,1.19)	−1.09 (−3.07, 0.89)
Baseline BMI				1.92 (1.62, 2.23)	1.73 (1.41, 2.06)***
Residence	Urban			Ref	
	Rural			−1.63 (−4.08, 0.81)	−1.95 (−4.48, 0.57)

Key: * = p<0.05 ** = p<0.01 *** = p<0.00.

ARV – Antiretroviral; BMI – Body mass Index; CD4 – Cluster of Differentiation 4; CI = Confidence Interval; TB – Tuberculosis; WHO – World Health Organization

**Model 2: model for measurement level variables only**: This model was developed by including the measurement level variables that were with p-value ≤ 0.25 during the bivariate analysis for adjustment. These variables were age, time, functional status, WHO clinical stage, TB infection status, TB prophylaxis status, other opportunistic infections, pain assessment, use of other medications than ARV, and ARV adherence. This model also showed that there was statistically significant variation in the weight progression of participants across individuals (var = 47.57, p < 0.001) (Table 3). The ICC calculated from this model indicated that 89.91% of the total variation in the mean weight of the participants could be attributed to inter-individual differences. The PCV for this model was calculated to be 16.39%. This indicated that 16.39% of the variation in the mean weight of participants between individuals was explained by the measurement level factors (Table 3).

**Table 3:** Random effects’ estimates for weight change among Human Immunodeficiency Virus (HIV) infected patients on anti-retroviral treatment in Mekelle hospital, Tigray, Ethiopia from January 2017 to December 2019 (n = 97 observations = 463).

Random effects				
	Null model	Measurement level variables only model	Individual level variables only model	Mixed model
	Estimate (95% CI)	Estimate (95% CI)	Estimate (95% CI)	Estimate (95% CI)
Var(constant)	56.31 (41.71, 76.02)	47.57 (35.00, 64.67)	12.53 (8.69, 18.07)	13.21 (9.03, 19.31)
Var(time)	4.38 (3.11, 6.16)	2.40 (1.64, 3.53)	5.00 (3.59, 6.95)	3.72 (2.58, 5.38)
Var residual	6.54 (5.50, 7.77)	5.31 (4.45, 6.34)	6.31 (5.34, 7.45)	5.35 (4.52, 6.34)
ICC in %	89.60	89.91	66.51	71.16
PCV	Reference	16.39	77.75	76.54
**Model selection**				
AIC	2742.439	2664.645	2629.937	**2564.329**

Key: AIC = Akaike Information Criteria; CI = Confidence Interval; ICC = Intraclass Correlation Coefficient; PCV = Percentage Change in Variance

**Model 3: Model adjustment for individual level variables only**: This model adjustment is for individual level variables which have a p-value ≤0.25 at the bivariate analysis. These are sex, religion, place of residence, baseline CD_4,_ baseline BMI, and viral load at six-month. This model showed that there was statistically significant variation in the mean weight of participants across individual (v_w_ = 12.53, p < 0.001). The ICC calculated from this model indicated that 66.51% of the total variability in the mean weight of participants was attributed to individual difference. The PCV for this model shows that above three-fourth (77.75%) of the variation in the mean weight of participants was explained by the individual level variables included in the model (Table 3).

**Model 4: Mixed model for measurement level and individual level factors**: This mixed model is a model developed for an adjustment of both the measurement and individual level variables simultaneously. The individual variation in the mean weight of participants has continued to be statistically significant (v_w_ = 13.21, p < 0.001). The ICC found from this mixed model showed that 71.16% of the total variance in mean weight of participants could be attributed to individual characteristics. A PCV of 76.54% implies that 76.54% of the variation in the mean weight of participants between individuals was explained by both measurement and individual variables included in the model (Table 3).

#### Fixed effects.

The fixed effects were explained using coefficients and their respective 95% CI.

**Measurement and individual level factors associated with weight progression**: The final mixed model best fits modeling weight progression of this sample as seen from the AIC result in which the mixed model has AIC value of 2564.326 and it is the lowest value from the other models’ AIC values. Hence interpretation of the factors associated with weight progression is based on the final mixed model output. In the final model where both measurement level and individual level factors were entered, sex of participant and baseline BMI were significant from the individual level factors, whereas age, time, tuberculosis infection status, tuberculosis prophylaxis status, the interaction term between tuberculosis infection status and tuberculosis prophylaxis status, and use of other medications than ARV were significant from the measurement level factors.

After controlling other measurement and individual level factors, the mean weight progression of females was 5.94 kg lower than males (β = −5.94, 95%CI [−7.40, −3.00]). Baseline BMI is also significant predictor of weight in which for a unit change in baseline BMI there is 1.92 kg progress in weight (β = 1.92 [1.41, 2.06]). The mean weight progression of participants aged from 35–44 years is 2.86 kg higher than participants aged from 15–24 years (β = 2.86 95% CI [0.29, 5.43]), and the mean weight progression of participants aged from 45–54 is 4.38 kg higher than participants aged 15–24 years (β = 4.38, 95% CI [0.63, 8.12]).

After controlling other measurement and individual level factors, the mean weight progression of participants at their 6^th^ month visit is 4.33 kg higher compared to their baseline measurement (β = 4.33 95% CI [3.13, 5.53]), the mean weight progression of participants at 12 month is 4.69 kg higher than their baseline weight measurement (β = 4.69, 95% CI [3.29, 6.09]), the mean weight progression of participants at 18 month is 5.17 kg higher than their baseline weight measurement (β = 5.17, 95% CI [3.57, 6.78]), and 24 month weight is 5.72 kg higher than the baseline measurement (β = 5.72, 95% CI [3.84, 7.61]).

After controlling other measurement and individual level factors, and taking participants who are not given tuberculosis prophylaxis as reference, tuberculosis infected participants are 12.04 kg lower in weight than those who are tuberculosis uninfected (β = −16.77, 95% CI [−21.92, −2.16]). Those participants who do not use other medications than ARV are 3.00 kg higher in weight compared to those participants who use other medications than ARV (β = 3.00, CI [0.14, 5.87]).

After controlling other measurement and individual level factors, the weight of tuberculosis uninfected and tuberculosis prophylaxis treated participants is 6.23 kg higher than those who are tuberculosis infected and tuberculosis prophylaxis untreated (β = 6.23, 95% CI [1.06, 11.40]) (Table 2).

### Weight progression among patients on ART and its’ predictors

The weight of patients on ART showed a gradual mean increase from the baseline up to 24 months of measurement significantly. The mean weight change was assessed at 6 months, 1 year, 18 month and 2 year with regard the baseline. On the sixth month of measurement patients gained a mean weight 3.08 kg with standard deviation of 4.86 kg compared to their baseline weight and this were significantly affected by baseline BMI, in which the normal weight (BMI 18.5–24.9 kg/m^2^) patients at baseline lost 1.89 kg (β = −1.89 CI [−3.11, −0.66]) compared with underweight patients at baseline. Weight gain varies between tuberculosis infection status groups in which patients who are tuberculosis infected at baseline gained 3.00 kg (β = 3.00, 95% CI [0.24, 5.76]) more at 6 months than those who were uninfected at baseline.

After one year ART follow up, patients gained a mean weight of 3.57 kg (SD = 6.05) and this was significantly determined by baseline BMI, CD4 count at baseline, WHO clinical stage, and ARV drug adherence (Table 4). Controlling the effects of other factors, after 12 month of ART follow-up patients who were normal weight at baseline lost 3.48 kg compared to those who were underweight at baseline (β = −3.48 CI [−4.48, −2.12]), patients who were overweight at baseline lost 3.59 kg compared with those who were underweight at baseline (β = −3.59 CI [−5.99, −1.19]), patients who were with CD4 count level >350 at baseline lost 1.64 kg (β = −2.94 CI [−2.94, −0.34]) compared with those who have CD4 count <=350. After 18 months ART follow up patients gained a mean weight 4.22 kg (SD = 7.10), and baseline BMI, CD4 count at baseline, WHO clinical stage, and ARV drug adherence were the significant factors (Table 4). Furthermore, the weight change after 2 years follow-up on ART was also assessed and the mean weight gain was 4.39 kg (SD = 7.45), and baseline BMI, CD4 count at baseline, WHO clinical stage, and ARV drug adherence were the significant factors (Table 4).

**Table 4:** Factors predicting weight gain at 6 months, 12 months, 18 month and 24 months compared to baseline weight among ART patients in Mekelle Hospital, Tigray, Ethiopia from January 2017 to December 2019 (n = 97, observations = 463).

Weight gain after 6 months ART follow up		
Factors		Β [95% CI)
Baseline BMI	Underweight (BMI < 18.5 kg/m^2^)	1
	Normal weight (BMI 18.5–24.9 kg/m^2^)	−1.89 [−3.11, −0.66]**
	Over weight (BMI 25–29.9 kg/m^2^)	−1.77 [−3.89, 0.36]
	Obese (BMI ≥ 30 kg/m^2^)	No obese
Tuberculosis infection status	No	1.00
	Yes	3.00 [0.24, 5.76]**
**Weight gain after 12 month ART follow up**		
Baseline BMI	Underweight (BMI < 18.5 kg/m^2^)	1.00
	Normal weight (BMI 18.5–24.9 kg/m^2^)	−3.48 [−4.84, −2.12]***
	Over weight (BMI 25–29.9 kg/m^2^)	−3.59 [−5.99, −1.19]**
	Obese (BMI ≥ 30 kg/m^2^)	No obese
CD_4_ count	<= 350	1.00
	>350	−1.64 [−2.94, −0.34]*
WHO clinical staging	Stage I	1.00
	Stage II	−1.00 [−2.68, 0.69]
	Stage III	−1.00 [−3.02, 1.02]
	Stage IV	−4.26 [−6.50, −2.02]***
Antiretroviral drug adherence	Good	1.00
	Fair	−4.16 [−10.95, 2.61]
	Poor	−3.10 [−5.40, −0.81]**
**Weight gain after 18 months ART follow up**		
Baseline BMI	Underweight (BMI < 18.5 kg/m^2^)	1.00
	Normal weight (BMI 18.5–24.9 kg/m^2^)	−4.24 [−6.03, −2.45]***
	Over weight (BMI 25–29.9 kg/m^2^)	−4.58 [−7.83, −1.34]**
	Obese (BMI ≥ 30 kg/m^2^)	No obese
CD_4_ count	<= 350	1.00
	>350	−2.45 [−4.17, −.73]**
WHO clinical staging	Stage I	1.00
	Stage II	−2.89 [−4.60, −1.18]**
	Stage III	−2.52 [−4.65, −0.39]*
	Stage IV	−5.56 [−7.87, −3.26]***
Antiretroviral drug adherence	Good	1.00
	Fair	−1.27 [−8.01, 5.46]
	Poor	−2.57 [−4.94, −0.21]*
**Weight gain after 24 month ART follow up**		
Baseline BMI	Underweight (BMI < 18.5 kg/m^2^)	1.00
	Normal weight (BMI 18.5–24.9 kg/m^2^)	−4.53 [−6.52, −2.54]***
	Over weight (BMI 25–29.9 kg/m^2^)	−5.02 [−8.65, −1.24]**
	Obese (BMI ≥ 30 kg/m^2^)	No obese
CD_4_ count	<= 350	1.00
	> 350	−2.53 [−4.43, −0.63]**
WHO clinical stage	Stage I	1.00
	Stage II	−3.50 [−5.01, −1.91]***
	Stage III	−2.74 [−4.72, −0.76]**
	Stage IV	−5.80 [−8.02, −3.58]***
Antiretroviral drug adherence	Good	1.00
	Fair	0.51 [−6.01, 7.02]
	Poor	−2.41 [−4.67, −0.16]*

Key: * = p<0.05 ** = p<0.01 *** = p<0.001.

ART – Antiretroviral; BMI – Body Mass Index; CI = Confidence Interval; CD4 – Cluster of Differentiation; WHO – World Health Organization

#### Model diagnostics results.

**Interaction effects**: Interaction terms were assessed among potential variables that can interact and no interaction term was found to be significant except the tuberculosis infection status*tuberculosis prophylaxis status interaction effect.

**Multi-collinearity**: Multi-collinearity was checked by using VIF. Therefore, there was no variable with VIF value of 10 or more indicating no multi-collinearity between the explanatory variables.

**Model selection:** AIC was used to select the model that best fits the data from all the four models built. Competitive models were compared and the model with lowest AIC value was selected. First of all, selection on the random intercept, random slope or both was performed. As shown below the random intercept and random slope model was selected having lowest AIC value.

## Discussion

The main aim of this paper was to determine weight progression over 2 years period and its’ predictors among patients on ART in Mekelle General Hospital, Tigray, Ethiopia. Understanding the fundamental but complex relationship between body weight and time and other factors for all stages of HIV disease is important in the clinical treatment of patients infected with HIV/AIDS. ARV treatment of HIV is correlated with a weight increase in this retrospective longitudinal study, in which HIV-positive patients followed for up to 2 years. Weight increases between the baseline measurements and consecutive measurements were observed among newly enrolled adult HIV patients in Mekelle General Hospital. This study revealed that sex and baseline BMI are individual level factors affecting weight of HIV-positive patients in Mekelle hospital, Tigray Ethiopia. In addition to the above individual level factors age of the participant, length of stay on ART, WHO clinical stage, tuberculosis infection status, ARV adherence, the interaction of tuberculosis infection status and tuberculosis prophylaxis status, and use of other medications than ARV are measurement level factors affecting weight of HIV patients in Mekelle General Hospital, Tigray, Ethiopia.

Weight measurement at the sixth month is significantly higher than the base line measurement, weight measurement at 12^th^ month of ART follow up is significantly higher than the baseline measurement, and weight measurement at 18^th^ month is also higher than the baseline measurement. The last weight measurement is again significantly higher than the baseline weight measurement. Regarding weight change at 6, 12, 18 and 24 months after baseline; tuberculosis infection status and baseline BMI were significant predictors of weight change at 6 months. While CD4 count, WHO clinical status, ARV drug adherence and baseline BMI were significant predictors of weight change at 12, 18 and 24 months.

Overall, the results show that more proportion of females were overweight at baseline compared to males (7.79% vs. 5%) which is in line with a study conducted in the United States [18,44]. Even though the proportion is higher in females than males, the proportion of females who are overweight in this study is quietly lower than the study conducted in the United States and it is also true for males, and this could be due to the economic difference between the study areas. From those who are underweight at baseline, 42.11% reached normal weight or higher at 6 months, 54.05% reached normal weight or higher at one year measurement and 18 months measurement (no difference between 1 year and 18 months measurement), and 64.9% of the underweight at baseline reached normal weight or higher at their 24-month measurement, and this is similar with a study conducted in the United States that showed 77% of those underweight at baseline progressed to normal weight or higher [22]. The mean weight change after 6 and 12-month of ART follow-up in this study is 3.07 kg and 3.57 kg respectively and this is comparably similar to two studies conducted in the United States [2,7] and higher than another study conducted in the United States [45]. Only 7.22% of the participants in this study were overweight or obese in this study at baseline, and this is quietly different when compared with a study conducted in Birmingham [46]. The reason for this difference could be the socio-economic differences between the study groups.

This study showed that age is a predicting factor for weight of HIV patients, and this is in line with studies conducted in Brazil, the United States, Tanzania and Ethiopia [18,28,47,48].

Length of stay on ART is another predictor variable that affects the weight of patients and this was in line with studies conducted in the United States and Ethiopia [2,28,49]. Patients with advanced WHO clinical stages have lower body weight in this study, and is similar to those conducted in Ethiopia [28,50].

This study shows that the factors associated with weight change after initiation of ART at 6 months include baseline BMI in which patients underweight at baseline gained more weight than other higher categories and this is similar to studies conducted in Brazil, the United States, Cambodia and Kenya that stated baseline BMI was strong predictor of weight gain [2,18,47,51]. Tuberculosis infection at baseline is another significant factor that determines weight gain in this study and it is in line with a study conducted in Jimma University Referral Hospital [49,52].

ARV drug adherence is a significant predictor of weight change after 12, 18, and 24 of ART follow-up in this study and this is in line with a study conducted in Vietnam [24].

There is a strong association between weight gain and CD4 lymphocyte count in this study which is in line with studies conducted in Vietnam, Ethiopia, and the United States [24,28,49,53] and patients with low CD4 count gained more weight than those with high CD4 count level at baseline which could be due to the boosting of the CD4 level is more efficient on low CD4 count than high CD4 count.

Weight gain was not different in sex groups in this study but a significant predictor in studies conducted in low socio-economic settings [54], and this could be due to a reason that higher proportion of females (79%) participated in this study.

The functional status of patients is not significant in this study and this contradicts with studies conducted in Ethiopia [28,50], and the reason for these differences could be the study participants in one of the studies conducted in Ethiopia were HIV patients on a second-line ART regimen. No association is found between weight gain and the presence of opportunistic infections in this study and this is in line with a study conducted in Tanzania [48]. Cotrimoxazole preventive therapy and IPT prophylaxis are not significant in this study but significant in a study conducted in Ethiopia [50], and this could be due to the study participants in the one study conducted in Ethiopia were HIV patients on second-line ART regimen.

At the last the effect of Hemoglobin was not examined in this study due to a high number of missing measurements in the records. In addition to this the effect of the ARV drug was again not controlled in this study following all the participants were TDF/3TC/EFZ regimen users.

### Limitations of the study

The main limitation of this study lies in its retrospective design; retrospective data analysis can only measure correlation, but no causality. Moreover, possible factors, like exercise and eating habits, economic status, alcohol drinking habit and smoking are not controlled in this analysis and could majorly influence weight. Due to its retrospective nature this study is not able to measure body composition. Additionally, full information on base line hemoglobin was not found for extraction with only 11(11.34%) records having hemoglobin level information.

## Conclusion and recommendation

In conclusion, this study suggested that both the measurement and individual level factors determine the progression of weight of study subjects in Mekelle General Hospital, Tigray, Ethiopia. Sex of study subjects, baseline BMI, length of stay on ART, WHO clinical stage, Tuberculosis infection status, ARV drug adherence, participant’s age, and use of medications other than ARV were significant predictors of weight progression. Regarding weight change, tuberculosis infection status and baseline BMI were the significant predictors at 6 months, and baseline BMI, WHO clinical stage, CD4 count and ARV drug adherence were predictors of weight change at 12 months, 18 months and 24 months. Therefore, interventions need to target at both the measurement and individual-level factors with special focus on predictor variables identified by the study.

## Supporting information

S1 File Ethical clearance. (TIF)

S2 File Final dataset. (DTA)

## References

[1] WHO I. Obesity and overweight. 2018.

[2] Crum-Cianflone N, Roediger MP, Eberly L, Headd M, Marconi V, Ganesan A, et al. Increasing rates of obesity among HIV-infected persons during the HIV epidemic. PLoS One. 2010;5(4):e10106. doi: 10.1371/journal.pone.0010106 20419086 PMC2856157

[3] WHO. HIV/AIDS key facts. Available from: https://www.who.int/news-room/fact-sheets/detail/hiv-aids. 2018.

[4] Range NS, Malenganisho W, Temu MM, Changalucha J, Magnussen P, Krarup H, et al. Body composition of HIV-positive patients with pulmonary tuberculosis: a cross-sectional study in Mwanza, Tanzania. Ann Trop Med Parasitol. 2010;104(1):81–90. doi: 10.1179/136485910X12607012373830 20149295

[5] Angel J, B B, Cooper R, Giguère P, Bream M, Boutilier A. A practical guide to HIV drug side effects. 2013:4–56.

[6] Koethe JR, Heimburger DC, PrayGod G, Filteau S. From wasting to obesity: the contribution of nutritional status to immune activation in HIV Infection. J Infect Dis. 2016;214 Suppl 2(Suppl 2):S75–82. doi: 10.1093/infdis/jiw286 27625434 PMC5021242

[7] Erlandson KM, Kitch D, Tierney C, Sax PE, Daar ES, Tebas P, et al. Weight and lean body mass change with antiretroviral initiation and impact on bone mineral density. AIDS. 2013;27(13):2069–79. doi: 10.1097/QAD.0b013e328361d25d 24384588 PMC3966569

[8] Norwood J, Turner M, Bofill C, Rebeiro P, Shepherd B, Bebawy S, et al. Brief report: weight gain in persons with HIV switched from efavirenz-based to integrase strand transfer inhibitor-based regimens. J Acquir Immune Defic Syndr. 2017;76(5):527–31. doi: 10.1097/QAI.0000000000001525 28825943 PMC5680113

[9] Health EFM o. National guidelines for comprehensive HIV prevention, care and treatment. 2017.

[10] Health UD, Services H, Panel on Antiretroviral Guidelines for Adults and Adolescents. Guidelines for the use of antiretroviral agents in adults and adolescents living with HIV. 2019.

[11] Kharsany ABM, Karim QA. HIV Infection and AIDS in Sub-Saharan Africa: current status, challenges and opportunities. Open AIDS J. 2016;10:34–48. doi: 10.2174/1874613601610010034 27347270 PMC4893541

[12] Ethiopia F o. Supplement to the 2014 National Comprehensive HIV Prevention, Care and Treatment Guideline of Ethiopia to Address HIV Test and Start. 2016.

[13] Organization WH. Global health sector strategy on HIV: 2016–2021: Towards ending AIDS. World Health Organization. 2016.

[14] WHO. Communities at the center, defending rights, breaking barriers and reaching with HIV services, global AIDS update. 2019.

[15] McCormick CL, Francis AM, Iliffe K, Webb H, Douch CJ, Pakianathan M, et al. Increasing obesity in treated female HIV patients from Sub-Saharan Africa: potential causes and possible targets for intervention. Front Immunol. 2014;5:507. doi: 10.3389/fimmu.2014.00507 25431572 PMC4230180

[16] Organization WH. Patient monitoring guidelines for HIV care and antiretroviral therapy (ART). 2006.

[17] Lakey W, Yang L-Y, Yancy W, Chow S-C, Hicks C. Short communication: from wasting to obesity: initial antiretroviral therapy and weight gain in HIV-infected persons. AIDS Res Hum Retroviruses. 2013;29(3):435–40. doi: 10.1089/aid.2012.0234 23072344 PMC3581041

[18] Yuh B, Tate J, Butt AA, Crothers K, Freiberg M, Leaf D, et al. Weight change after antiretroviral therapy and mortality. Clin Infect Dis. 2015;60(12):1852–9. doi: 10.1093/cid/civ192 25761868 PMC4542664

[19] Sharma A, Bynum SA, Schneider MF, Cox C, Tien PC, Hershow RC, et al. Changes in body mass index following HAART initiation among HIV-Infected women in the women’s interagency HIV study. J AIDS Clin Res. 2014;5:1000323. doi: 10.4172/2155-6113.1000323 25580365 PMC4285631

[20] Hasse B, et al. Obesity trends and body mass index changes after starting antiretroviral treatment: the Swiss HIV Cohort Study. Open Forum Infectious Diseases. 2014. 10.1093/ofid/ofu040 PMC4281814 25734114

[21] Koethe JR, Heimburger DC. Nutritional aspects of HIV-associated wasting in sub-Saharan Africa. Am J Clin Nutr. 2010;91(4):1138S-1142S. doi: 10.3945/ajcn.2010.28608D 20147470 PMC2844686

[22] Corless IB, Nicholas PK, McGibbon CA, Wilson C. Weight change, body image, and quality of life in HIV disease: a pilot study. Appl Nurs Res. 2004;17(4):292–6. doi: 10.1016/s0897-1897(04)00076-x 15573338

[23] McComsey GA, Moser C, Currier J, Ribaudo HJ, Paczuski P, Dubé MP, et al. Body composition changes after initiation of raltegravir or protease inhibitors: ACTG A5260s. Clin Infect Dis. 2016;62(7):853–62. doi: 10.1093/cid/ciw017 26797215 PMC4787610

[24] Tang AM, Sheehan HB, Jordan MR, Duong DV, Terrin N, Dong K, et al. Predictors of weight change in male HIV-positive injection drug users initiating antiretroviral therapy in Hanoi, Vietnam. AIDS Res Treat. 2011;2011:890308. doi: 10.1155/2011/890308 21776380 PMC3137978

[25] Guo Y, Logan HL, Glueck DH, Muller KE. Selecting a sample size for studies with repeated measures. BMC Med Res Methodol. 2013;13:100. doi: 10.1186/1471-2288-13-100 23902644 PMC3734029

[26] Bureau TRH. Tigray Regional Health Bureau 2010 EFY Annual Report. 2018.

[27] Hoffman L. Longitudinal analysis: Modeling within-person fluctuation and change. Routledge. 2015.

[28] Reda AA, Biadgilign S, Deribew A, Gebre B, Deribe K. Predictors of change in CD4 lymphocyte count and weight among HIV infected patients on anti-retroviral treatment in Ethiopia: a retrospective longitudinal study. PLoS One. 2013;8(4):e58595. doi: 10.1371/journal.pone.0058595 23573191 PMC3616015

[29] Ethiopia, FDR.o. National guidelines for comprehensive hiv prevention, care and treatment. 2014.

[30] Phan J, Reue K. Lipin, a lipodystrophy and obesity gene. Cell Metab. 2005;1(1):73–83. doi: 10.1016/j.cmet.2004.12.002 16054046

[31] Ibrahim JG, Molenberghs G. Missing data methods in longitudinal studies: a review. Test (Madr). 2009;18(1):1–43. doi: 10.1007/s11749-009-0138-x 21218187 PMC3016756

[32] Rabe-Hesketh S. Multilevel and longitudinal modeling using Stata. STATA press. 2008.

[33] Raudenbush SW. Hierarchical linear models: Applications and data analysis methods. Advanced Quantitative Techniques in the Social Sciences Series/SAGE. 2002.

[34] Hox JJ. Multilevel analysis: techniques and applications. 2 ed. New York: Routledge. 2010.

[35] Berridge D, Crouchley R, Grose D. Multivariate generalized linear mixed models using R. Boca Raton, FL: CRC press. 2011.

[36] Verbek G, M G. Linear Mixed Models for Longitudinal Data. Springer. 2000:579.

[37] Çöltekin Ç. Multilevel (or mixed-effect) linear models. 2013.

[38] West BT, Welch KB, Galecki AT. Linear mixed models: a practical guide using statistical software. Chapman and Hall/CRC. 2022.

[39] Muller KE, Stewart PW. Linear model theory: univariate, multivariate, and mixed models. John Wiley & Sons. 2006.

[40] Faraway JJ. Extending the linear model with R: generalized linear, mixed effects and nonparametric regression models. Chapman and Hall/CRC. 2016.

[41] Galecki A, T B. Linear mixed effects models using R, step by step approach. Springer Texts in Statistics. 2013.

[42] Pituch KA. Applied multivariate statistics for the social sciences. Francis T. 2016.

[43] Luke D. Multilevel modeling. Thousand Oaks: Sage Publications. 2004.

[44] Bares SH, Smeaton LM, Xu A, Godfrey C, McComsey GA. HIV-infected women gain more weight than HIV-infected men following the initiation of antiretroviral therapy. J Womens Health (Larchmt). 2018;27(9):1162–9. doi: 10.1089/jwh.2017.6717 29608129 PMC6148723

[45] Taramasso L., et al. Factors associated with weight gain in people treated with dolutegravir. in Open Forum Infectious Diseases. 2020. Oxford University Press US. 10.1093/ofid/ofaa195 PMC7295329 32577427

[46] Tate T, Willig AL, Willig JH, Raper JL, Moneyham L, Kempf M-C, et al. HIV infection and obesity: where did all the wasting go?. Antivir Ther. 2012;17(7):1281–9. doi: 10.3851/IMP2348 22951353 PMC3779137

[47] Maia Leite LH, De Mattos Marinho Sampaio AB. Progression to overweight, obesity and associated factors after antiretroviral therapy initiation among Brazilian persons with HIV/AIDS. Nutr Hosp. 2010;25(4):635–40. 20694301

[48] Li N, Spiegelman D, Drain P, Mwiru RS, Mugusi F, Chalamilla G, et al. Predictors of weight loss after HAART initiation among HIV-infected adults in Tanzania. AIDS. 2012;26(5):577–85. doi: 10.1097/QAD.0b013e32834f9851 22156968

[49] Hadgu TH, et al. Undernutrition among HIV positive women in Humera hospital, Tigray, Ethiopia: antiretroviral therapy alone is not enough, cross sectional study. BMC Public Health. 2013;13:1–10. 24107008 10.1186/1471-2458-13-943 PMC3852443

[50] Baraki AG, Gezie LD, Zeleke EG, Awoke T, Tsegaye AT. Body mass index variation over time and associated factors among HIV-positive adults on second-line ART in north-west Ethiopia: a retrospective follow-up study. BMJ Open. 2019;9(9):e033393. doi: 10.1136/bmjopen-2019-033393 31551394 PMC6773344

[51] Madec Y, Szumilin E, Genevier C, Ferradini L, Balkan S, Pujades M, et al. Weight gain at 3 months of antiretroviral therapy is strongly associated with survival: evidence from two developing countries. AIDS. 2009;23(7):853–61. doi: 10.1097/QAD.0b013e32832913ee 19287299

[52] Filate M, Mehari Z, Alemu YM. Longitudinal body weight and sputum conversion in patients with tuberculosis, Southwest Ethiopia: a retrospective follow-up study. BMJ Open. 2018;8(9):e019076. doi: 10.1136/bmjopen-2017-019076 30185566 PMC6129038

[53] Mwamburi DM, Wilson IB, Jacobson DL, Spiegelman D, Gorbach SL, Knox TA, et al. Understanding the role of HIV load in determining weight change in the era of highly active antiretroviral therapy. Clin Infect Dis. 2005;40(1):167–73. doi: 10.1086/426591 15614708

[54] Huisin ʼt Veld D, Balestre E, Buyze J, Menten J, Jaquet A, Cooper DA, et al. Determinants of weight evolution among HIV-positive patients initiating antiretroviral treatment in low-resource settings. J Acquir Immune Defic Syndr. 2015;70(2):146–54. doi: 10.1097/QAI.0000000000000691 26375465 PMC4576726

