# Supplementary figures and images for: Predictors of weight progression among HIV infected adults on anti-retroviral treatment in Mekelle hospital, Tigray, Ethiopia: A longitudinal study
Source: PLoS One. 2025 Jul 18;20(7):e0327392. doi: 10.1371/journal.pone.0327392 (PMC12273959; doi:10.1371/journal.pone.0327392)

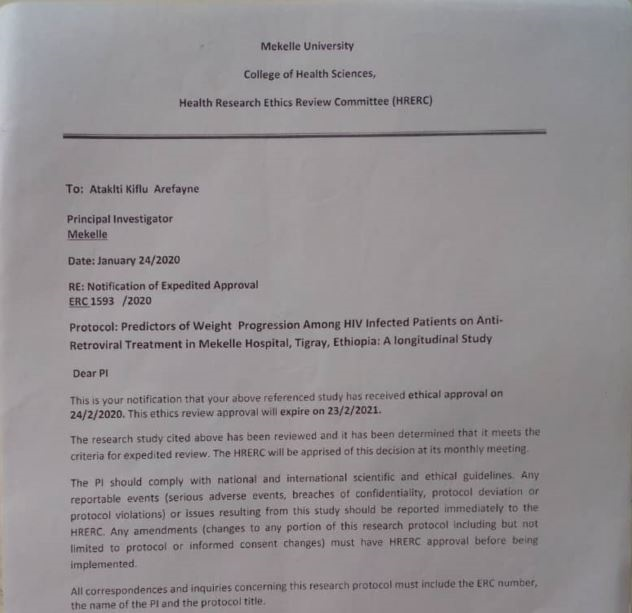

Supplement: S1 File — (TIF) [file pone.0327392.s001.tif]
